# The Role of Monocyte to High-Density Lipoprotein Cholesterol Ratio in Predicting the Severity of Proteinuria and Renal Dysfunction in Primary Nephrotic Syndrome

**DOI:** 10.7759/cureus.20345

**Published:** 2021-12-11

**Authors:** Fatma Yılmaz Aydın, Eren Eynel, İdris Oruç, Hasan Ince, Enver Yüksel, Emre Aydın

**Affiliations:** 1 Internal Medicine, Dicle University, Diyarbakır, TUR; 2 Nephrology, Dicle University, Diyarbakır, TUR; 3 Nephrology, Gazi Yaşargil Education and Research Hospital, Diyarbakır, TUR

**Keywords:** renal function, inflammation, proteinuria, monocytes to high density lipoprotein cholesterol ratio, nephrotic syndrome

## Abstract

Introduction: Monocyte to high-density lipoprotein (HDL) cholesterol ratio (MHR) has emerged as a novel marker of endothelial injury, inflammation, and oxidative stress. This study aimed to investigate the effect of MHR on primary nephrotic syndrome (NS) and its relationship with the severity of proteinuria.

Methods: This study enrolled 161 patients newly diagnosed with primary NS and 100 healthy individuals. Demographic characteristics of the patients, primary NS diagnosis, basal laboratory parameters, the amount of 24-hour urinary protein excretion, and MHR were recorded. The groups were compared regarding these parameters.

Results: MHR was significantly higher in patients with primary NS compared with the healthy group (12.89 ± 4.86 and 9.71 ± 2.30, respectively; p < 0.001). There was no difference between the groups in terms of age and sex. The amount of protein in the 24-hour urine in patients with a diagnosis of primary NS was 6.91 ± 3.73 g/day. The correlation analysis showed a positive correlation between MHR and the amount of proteinuria (r = 0.519, p < 0.001) and creatinine level (r = 0.167, p = 0.034). The multivariate regression analysis found that the severity of proteinuria was independently correlated to MHR (p < 0.001). According to the receiver operating characteristic curve analysis, the optimal cut-off level for MHR in NS was 10.08 (area under the curve of 0.704, sensitivity of 68%, and a specificity of 62%).

Conclusion: Our study is the first to compare the severity of proteinuria and renal functions with MHR in patients with primary NS. We believe that MHR can be used as a biomarker to determine inflammation, endothelial injury, and the level of oxidative stress, and may be useful to predict prognosis in patients with primary NS.

## Introduction

Nephrotic syndrome (NS) is a disorder that causes proteinuria as a result of increased permeability of the glomerular filtration membrane [1]. Its pathophysiology involves injury to the filtration barrier, which is composed of the glomerular basement membrane, podocytes, and glomerular endothelium [2]. In NS, there is a proinflammatory state associated with high tumor necrosis factor α, interleukin 6, and fibrinogen levels [3]. Furthermore, it has been shown that NS is associated with endothelial dysfunction [4], and as the disease enters remission, endothelial injury improves [5]. Additionally, increased oxidative stress is associated with glomerular injury, resulting in chronic renal injury [6,7].

Researchers all over the world have focused on discovering reproducible, simple, fast, and low-cost biomarkers to evaluate endothelial dysfunction and to determine inflammatory status and oxidative stress. Various cytokines are released in response to endothelial dysfunction, inflammation, and increased oxidative stress. Monocytes are the first cells that are activated in this process. Therefore, they are of critical importance for inflammation. Activated monocytes are transformed into macrophages. These monocytes and macrophages phagocytes oxidize low-density lipoprotein (LDL) cholesterol molecules and form foam cells, thereby inducing the release of pro-inflammatory and pro-oxidant cytokines. High-density lipoprotein (HDL) cholesterol, on the other hand, reduces monocyte activation and protects endothelium against the detrimental effects of oxidized LDL cholesterol particles. Moreover, HDL prevents the expression of adhesion molecules and causes nitric oxide release, and induces vasodilation. Due to these properties, it has anti-inflammatory and anti-oxidant properties. An increased count of pro-inflammatory monocytes and a reduced level of anti-inflammatory HDL cholesterol have been considered novel markers of inflammation. As a result of these studies, the monocyte to HDL cholesterol ratio (MHR) has emerged as a novel marker for determining endothelial injury, inflammation, and oxidative stress [8-12].

Although the relationship of MHR, a new biomarker, and many diseases where chronic inflammation is involved has been evaluated, no study related to primary NS has been performed. In the present study, we aimed to investigate the influence of MHR on primary NS and its relationship with the severity of proteinuria.

## Materials and methods

This study was conducted with a total of 261 subjects. The study population consisted of newly diagnosed primary NS patients of both sexes older than 18 years of age, who presented to the nephrology and internal medicine outpatient clinics between January 2017 and December 2020, and healthy controls. A total of 161 patients with newly diagnosed primary NS and 100 healthy subjects were enrolled. Patients who were under the age of 18 years, who had secondary NS, active infection, chronic inflammatory disease, definitive or suspected malignancy, hematological disorders, or treated hyperlipidemia were excluded. The control group consisted of healthy individuals free of any documented disease, who presented to the internal medicine outpatient clinic; the NS group was composed of patients with newly diagnosed primary NS. The demographic properties (age and sex), primary NS diagnosis, biochemical parameters at the time of diagnosis (creatinine, albumin, total protein, LDL, and HDL), C-reactive protein, complete blood count parameters, amount of 24-hour proteinuria, and MHR were recorded in all patients. MHR was calculated as the ratio of monocyte count to the HDL cholesterol level. The same parameters were also evaluated in the control group, and both groups were compared with regard to these parameters.

Statistical analysis

Study data were statistically analyzed using Statistical Package for Social Sciences (SPSS) version 24.0 (SPSS Inc., Chicago, IL). The normality of the distribution of the study variables was tested with visual (histogram and likelihood graphics) and analytic methods (Kolmogorov-Smirnov/Shapiro-Wilk tests). The descriptive analyses were presented as mean and standard deviation for normally distributed variables and median and min-max values for non-normally distributed variables. Inter-group comparisons were carried out using Student's t-test for normally distributed variables and Mann-Whitney U test for non-normally distributed ones. The chi-square test was used to test the difference in frequencies between the groups. Correlation between continuous variables was tested with Pearson and Spearman correlation analysis tests, depending on the normality of distribution. A linear regression analysis was performed to determine factors affecting proteinuria. The diagnostic decision-making properties of the MHR value in predicting patients with primary NS were analyzed with the receiver operating characteristics (ROC) curve. When a significant cut-off value was observed, the sensitivity and specificity values were presented. A p-value of less than 0.05 was considered statistically significant.

## Results

A total of 261 patients were enrolled in our study, 161 of whom were primary NS patients and 100 were healthy controls. Of these, 129 (49.4%) were women, and 132 (50.6%) were men. The mean age of the total study population was 35.92 ± 11.94 years.

**Figure 1:**
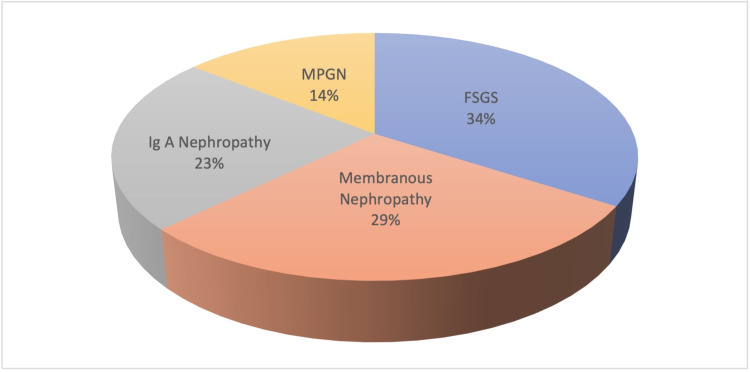
Diagnoses of primary nephrotic syndrome. IgA: immunoglobulin A; MPGN: membranoproliferative glomerulonephritis; FSGS: focal segmental glomerulosclerosis.

The primary NS group had a mean age of 36.04 ± 12.54 years, and it consisted of 75 (46.6%) women and 86 (53.4%) men. There was no significant difference between the study groups regarding age or sex (p = 0.832 and p = 0.244, respectively). The etiology of primary NS was focal segmental glomerulosclerosis (FSGS) in 34% of patients, membranous glomerulonephritis in 29%, immunoglobulin A (IgA) nephropathy in 23%, and membranoproliferative glomerulonephritis (MPGN) in 14% (Figure 1). An analysis of the laboratory parameters at the time of diagnosis in the primary NS group showed that the mean amount of 24-hour proteinuria was 6.91 ± 3.73, mean albumin level was 2.81 ± 0.65, mean LDL level was 156.39 ± 87.15, mean HDL level was 43.90 ± 10.08, and mean monocyte count was 544.04 ± 188.61. MHR was calculated as 12.89 ± 4.86. A comparison of laboratory parameters of the primary NS group with those of the healthy group showed that, as compared with the controls, the NS group had a significantly higher serum creatinine, LDL, HDL, C-reactive protein (CRP), neutrophil count, monocyte count, and MHR but significantly lower albumin and total protein levels. Although hemoglobin level, thrombocyte count, and lymphocyte count were lower in the primary NS group, the differences did not reach statistical significance. The demographic and laboratory data of the study groups are shown in Table 1.

**Table 1:** Demographic and laboratory characteristics of the groups. LDL: low-density lipoprotein; HDL: high-density lipoprotein; CRP: C-reactive protein; MHR: monocyte to high-density lipoprotein cholesterol ratio.

Parameters	Primary nephrotic syndrome group (n = 161)	Control group (n = 100)	p-value
Age	36.04 ± 12.54	35.72 ± 10.97	0.832
Sex (female, male)	75 (%46.6), 86 (%53.4)	54 (%54), 46 (%46)	0.244
Creatinine (mg/dL)	1.48 ± 1.08	0.72 ± 0.14	<0.001
Albumin (g/dL)	2.81 ± 0.65	4.35 ± 0.30	<0.001
Total protein (g/dL)	5.74 ± 1.26	7.37 ± 0.39	<0.001
LDL (mg/dL)	156.39 ± 87.15	109.86 ± 23.35	<0.001
HDL (mg/dL)	43.90 ± 10.08	48.37 ± 7.85	<0.001
CRP (mg/L)	6.57 ± 7.78	3.62 ± 0.53	<0.001
Hemoglobin (g/dL)	13.16 ± 2.69	13.50 ± 0.65	0.206
Neutrophil (10^3^/mL)	5505.96 ± 2162.72	3984.80 ± 1078.60	<0.001
Lymphocyte (10^3^/mL)	2248.88 ± 780.99	2303.0 ± 529.49	0.542
Monocyte (10^3^/mL)	544.04 ± 188.61	460.73 ± 88.42	<0.001
Platelet (10^9^/mL)	259.51 ± 82.31	274.14 ± 48.01	0.108
24-h proteinuria (g/day)	6.91 ± 3.73	0	<0.001
MHR	12.89 ± 4.86	9.71 ± 2.30	<0.001

**Figure 2:**
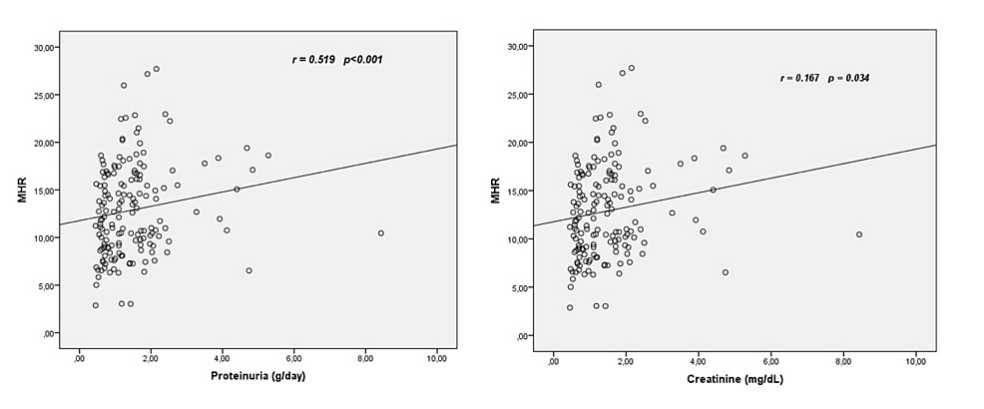
Correlation analysis between MHR and amount of proteinuria and serum creatinine levels. MHR: monocyte to high-density lipoprotein cholesterol ratio.

A correlation analysis between MHR, proteinuria, and other parameters in the primary NS group was carried out using the Pearson correlation test (Table 2). There was no significant correlation between MHR and age, lymphocyte count, neutrophil count, thrombocyte count, and hemoglobin level. In contrast, MHR showed a significant positive correlation to proteinuria, creatinine, LDL, CRP, and monocyte count, and a significant negative correlation to serum albumin, total protein, and HDL level. A correlation analysis with proteinuria showed that the amount of proteinuria showed a significant positive correlation to MHR, monocyte count, and LDL level, and a significant negative correlation to serum albumin and total protein (Table 2). The correlation analysis between MHR and the amount of proteinuria and serum creatinine level is shown in Figure 2.

**Table 2:** Correlation analysis of MHR and proteinuria with other parameters. MHR: monocyte to high-density lipoprotein cholesterol ratio; LDL: low-density lipoprotein; HDL: high-density lipoprotein; CRP: C-reactive protein.

	MHR		Proteinuria	
Parameters	r	p	r	p
Age	0.063	0.424	0.031	0.699
Proteinuria/MHR	0.519	<0.001	0.519	<0.001
Creatinine	0.167	0.034	0.084	0.287
Albumin	−0.209	0.008	−0.427	<0.001
Total protein	−0.205	0.009	−0.395	<0.001
LDL	0.222	0.005	0.289	<0.001
HDL	−0.448	<0.001	−0.152	0.054
Monocytes	0.813	<0.001	0.486	<0.001
Lymphocyte	0.137	0.084	0.159	0.044
Neutrophil	−0.080	0.316	−0.046	0.562
Platelet	0.129	0.104	0.097	0.221
Hemoglobin	−0.098	0.217	0.018	0.823
CRP	0.174	0.027	0.078	0.326

The univariate and multivariate regression analyses were performed to confirm the independent predictors of the severity of proteinuria (Table 3). In the univariate regression analysis, serum albumin, total protein, LDL, monocyte count, lymphocyte count, and MHR were found to be correlated to the severity of proteinuria. In the multivariate regression analysis, the severity of proteinuria was independently correlated to serum albumin and MHR (p = 0.004 and p < 0.001, respectively).

**Table 3:** Univariate and multivariate linear regression analysis of the proteinuria. HDL: high-density lipoprotein; LDL: low-density lipoprotein; CRP: C-reactive protein; MHR: monocyte to high-density lipoprotein cholesterol ratio.

	Univariate				Multivariate			
Independent variables	Odds ratio	95% CI		p-value	Odds ratio	95% CI		p-value
Age	0.01	−0.03	0.06	0.699				
Creatinine	0.29	−0.25	0.83	0.287				
Albumin	−2.44	−3.25	−1.63	<0.001	−1.32	−2.20	−0.43	0.004
Total protein	−1.17	−1.59	−0.74	<0.001	−0.51	−1.04	0.03	0.062
HDL	−0.06	−0.11	0.001	0.054				
LDL	0.01	0.01	0.02	<0.001	−0.001	−0.01	0.01	0.794
Monocytes	0.01	0.007	0.012	<0.001	0.002	−0.003	0.006	0.434
Lymphocyte	0.76	0.02	1.50	0.044	0.32	−0.28	0.92	0.291
Neutrophil	−0.08	−0.35	0.19	0.562				
Platelet	0.004	−0.01	0.01	0.221				
Hemoglobin	0.03	−0.12	0.24	0.823				
CRP	0.04	−0.38	−0.11	0.326				
MHR	0.40	0.30	0.50	<0.001	0.28	0.11	0.44	<0.001

**Figure 3:**
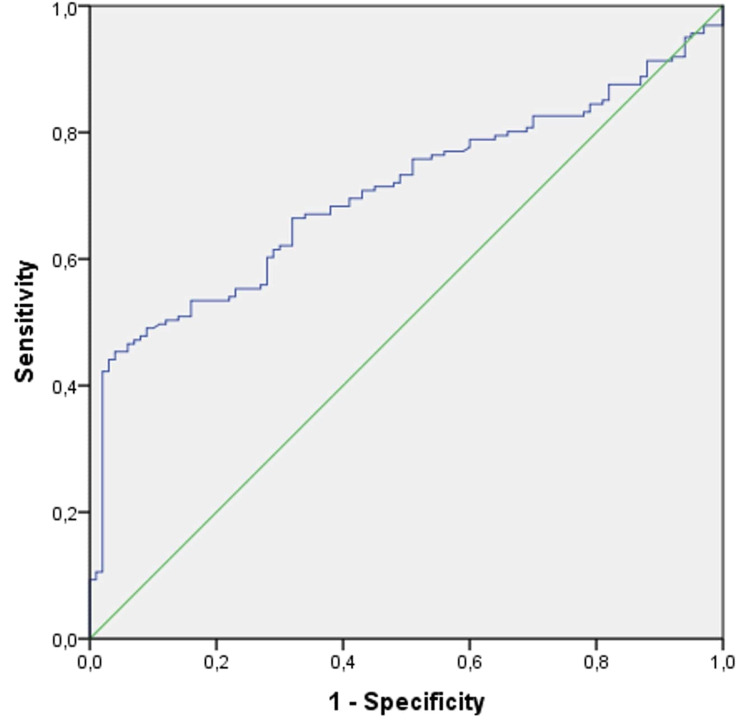
Receiver operating characteristic curve analysis of MHR. MHR: monocyte to high-density lipoprotein cholesterol ratio.

According to the ROC curve analysis, the optimal cut-off value of MHR in NS was found at 10.08. MHR had an AUC of 0.704 (95% CI, 0.642-0.766, p < 0.001), with a sensitivity of 68% and a specificity of 62% (Figure 3).

## Discussion

In our study, we investigated the effect of MHR on primary NS and its relationship with the severity of proteinuria. We found that MHR significantly increased in the primary NS group compared with the healthy control group. We detected a positive correlation between MHR and the severity of proteinuria and serum creatinine level. Our study is important in that it is the first study in the literature to examine the relationship of MHR with the severity of proteinuria in patients with primary NS.

A review of the current literature indicates that many studies to date have shown that MHR, which is calculated by the ratio of these two parameters, which are known to be closely associated with endothelial dysfunction and inflammation, can be a new marker of inflammation and oxidative stress in systemic diseases, while it is also associated with the prognosis of some diseases. It is suggested that increased MHR level predicts disease progression [13].

In a study conducted with 262 diabetes mellitus (DM) patients, it was found that MHR was significantly higher in the DM group compared with the healthy group. In that study, when the subjects were grouped as those with and without nephropathy, MHR was significantly higher in those who had nephropathy [14]. A separate study that analyzed the relationship between DM and MHR found a significantly higher MHR in DM patients [15]. Three separate studies that evaluated metabolic syndrome and MHR have determined that MHR significantly increased in patients with metabolic syndrome, and MHR could be an independent predictor of metabolic syndrome [13,16,17]. MHR was also studied in hypertensive (HT) patients in whom the endothelial injury is involved; MHR was found to be significantly increased in that population compared with healthy controls. Increased MHR was considered an independent risk factor in HT patients [11,13]. The above-mentioned studies have indicated that the MHR level is increased by many diseases, in the pathophysiology of which endothelial injury, inflammation, and oxidative stress play a role. In accordance with the literature, we found a significantly higher MHR level in patients with primary NS caused by these factors as compared with the healthy controls (p < 0.001).

Additionally, we evaluated renal function based on serum creatinine level in patients with primary NS and analyzed its correlation with MHR. We detected a positive correlation between MHR and serum creatinine level (r = 0.167, p = 0.034). There are several studies in the literature which have examined the relationship between renal functions and MHR. In a study where 8,159 patients were analyzed, the effect of reduced renal function on MHR was evaluated. That study found a significantly higher MHR level in patients with reduced renal function [18]. Kanbay et al. and Gembillo et al. demonstrated that MHR increased in parallel to estimated glomerular filtration rate (eGFR) reduction [19,20]. Our results show similarity with those studies.

Proteinuria is a diagnostic marker of NS while it also affects disease progression [21]. When NS remains untreated, glomerular injury progresses, the severity of proteinuria increases, and these events are accompanied by reduced renal function. As a result, end-stage renal failure may ensue in patients. Thus, the severity of proteinuria is an important clinical parameter that affects patient morbidity and mortality. Therefore, many studies have been conducted to investigate proteinuria, which has a significant role in renal injury. Karataş et al. determined that as the amount of proteinuria increased, MHR also increased in the DM nephropathy group, suggesting that elevated MHR may be a biomarker for DM nephropathy [22]. Kaplan et al. examined the role of MHR in predicting the complications of hypertension. They found significantly more severe proteinuria and a significantly higher MHR in the non-dipper group than the dipper HT group [23]. Other studies on HT patients have revealed a positive correlation between the severity of proteinuria and MHR [10,11]. These studies have suggested that MHR could be an independent determinant of urinary protein excretion. In line with the studies mentioned above, our study demonstrated that MHR showed a positive correlation to the severity of proteinuria, with its level being increased as the severity of proteinuria increased (r = 0.519, p < 0.001). Furthermore, when we evaluated the regression analysis, we determined that the severity of proteinuria was independently correlated to MHR (p < 0.001).

## Conclusions

In conclusion, our study is the first to compare the severity of proteinuria and renal functions with MHR. Our results revealed that increased MHR levels were significantly and independently correlated to the severity of proteinuria. We determined a positive correlation between serum creatinine and MHR. We believe that MHR can be used as a biomarker to detect inflammation, endothelial injury, and oxidative stress level and prove useful to predict prognosis in primary NS patients.
